# Endocytic protein Pal1 regulates appressorium formation and is required for full virulence of *Magnaporthe oryzae*

**DOI:** 10.1111/mpp.13149

**Published:** 2021-10-12

**Authors:** Deng Chen, Hong Hu, Wenhui He, Shimei Zhang, Mengxi Tang, Shikun Xiang, Caiyun Liu, Xuan Cai, Ahmed Hendy, Muhammad Kamran, Hao Liu, Lu Zheng, Junbing Huang, Xiao‐Lin Chen, Junjie Xing

**Affiliations:** ^1^ State Key Laboratory of Hybrid Rice Hunan Hybrid Rice Research Center Changsha China; ^2^ State Key Laboratory of Agricultural Microbiology and Provincial Key Laboratory of Plant Pathology of Hubei Province College of Plant Science and Technology Huazhong Agricultural University Wuhan China

**Keywords:** actin ring, appressorium formation, autophagy, cAMP pathway, endocytosis, Pmk1 pathway

## Abstract

Endocytosis plays key roles during infection of plant‐pathogenic fungi, but its regulatory mechanisms are still largely unknown. Here, we identified a putative endocytosis‐related gene, *PAL1*, which was highly expressed in appressorium of *Magnaporthe oryzae*, and was found to be important for appressorium formation and maturation. Deletion of *PAL1* significantly reduced the virulence of *M. oryzae* due to defects in appressorial penetration and invasive growth in host cells. The Pal1 protein interacted and colocalized with the endocytosis protein Sla1, suggesting it is involved in endocytosis. The Δ*pal1* mutant was significantly reduced in appressorium formation, which was recovered by adding exogenous cAMP and 3‐isobutyl‐1‐methylxanthine (IBMX). Moreover, the phosphorylation level of Pmk1 in Δ*pal1* was also reduced, suggesting Pal1 functions upstream of both the cAMP and Pmk1 signalling pathways. As a consequence, the utilization of glycogen and lipid, appressorial autophagy, actin ring formation, localization of septin proteins, as well as turgor accumulation were all affected in the Δ*pal1* mutant. Taken together, Pal1 regulates cAMP and the Pmk1 signalling pathway for appressorium formation and maturation to facilitate infection of *M. oryzae*.

## Graphical abstract

Endocytic protein Pal1 of *Magnaporthe oryzae* contributes to full pathogenicity by regulating cAMP and the Pmk1 signalling pathway for appressorium formation and maturation.

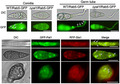

## 1 INTRODUCTION

The ascomycete fungus *Magnaporthe oryzae* is the causal agent of rice blast, which has become a model organism for studying plant–pathogen interactions (Dean et al., 2012; Ebbole, 2007). During infection, *M. oryzae* forms a melanized appressorium on the host surface, breaches the host cuticle with a penetration peg, and spreads across host cells through invasive hyphae (Ebbole, 2007; Wilson & Talbot, 2009). The formation and maturation of the appressorium is a key and complex process that is determined by spore storage components (such as glycogen and lipid) and is regulated by a variety of signalling pathways (Rho et al., 2009; Talbot, 2003).

The cAMP signalling pathway is one of the most important signalling pathways in the formation of the appressorium in *M. oryzae* (Adachi & Hamer, 1998; Mitchell & Dean, 1995; Xu & Hamer, 1996). Previous studies have confirmed that intracellular cAMP concentration gradually increases during appressorium differentiation of *M. oryzae* (Lee & Dean, 1993). cAMP acts as a second messenger in cells, transmitting intracellular and extracellular signals to regulate the formation and differentiation of the appressorium (Lee & Dean, 1993; Xu & Hamer, 1996). The Pmk1–MAPK signalling pathway is another pathway that participates in the late stage of appressorium formation, called appressorium maturation. This pathway consists of the Mst11–Mst7–Pmk1 protein kinase cascade (Wilson & Talbot, 2009; Xu & Hamer, 1996). Although the key components of cAMP and the Pmk1–MAPK signalling pathway have been identified, their upstream regulatory mechanism in *M. oryzae* is still unclear. Mucin protein MoMsb2 is responsible for cell surface recognition, is required for Pmk1 phosphorylation, and may also play a role upstream of the cAMP signalling pathway (Liu et al., 2011; Wang et al., 2015).

In *M. oryzae*, during the formation and maturation of the appressorium, a series of biological processes are activated, so that the appressorium has all the functions needed to ensure successful invasion and colonization (Foster et al., 2017; Rebollar & López‐García, 2013). These processes include glycogen, lipid, and trehalose utilization (Foster et al., 2017; Jong et al., 1997; Thines et al., 2000), peroxisome biogenesis (Chen et al., 2017), the autophagy process (Kershaw & Talbot, 2009; Veneault‐Fourrey et al., 2006), endocytosis, actin ring formation, and melanin synthesis (Li, Chen et al., 2017), as well as turgor accumulation (Jong et al., 1997). In addition to the strong turgor effect, the appressorium also needs enough mechanical pressure to produce the penetration peg, which is provided by the appressorium actin ring formed in the middle of the contact between the mature appressorium and the host (Li, Gao et al., 2017). The appressorium actin ring is assembled by septin proteins, which are composed of Sep3, Sep4, Sep5, and Sep6 (Dagdas et al., 2012; Ryder et al., 2013).

Endocytosis is a complex cellular process that mainly includes cargo sorting, membrane invagination, vesicle scission, and targeting (Kaksonen et al., 2005). The first and most interesting step of endocytosis is site formation or establishment (Carroll et al., 2012; Toret & Drubin, 2006). In budding yeast, dozens of proteins participate in this step, including early coat, WASP/Myo, and amphiphysin (Carroll et al., 2012; Kaksonen et al., 2005; Munn, 2000). The recruitment of proteins to endocytic sites is carried out in a highly precise order. In *Saccharomyces cerevisiae*, Ede1p, Syp1p, clathrin, Pal1p (Pal1 domain‐containing protein), and the AP2 complex are the first proteins to arrive at nascent endocytic sites (Carroll et al., 2012; Ge et al., 2005; Newpher et al., 2005). Proteins containing clathrin, AP2 complex, yap1801p, yap1802p, and Pal1p work as a new early coat module and internalize with vesicles before disassembly (Carroll et al., 2012; Sun et al., 2006). In *M. oryzae*, several endocytosis genes, such as *MoEND3*, *MoABP1*, and *MoARK1*, have been identified to play important roles in receptor internalization, signalling pathway activation, cytoskeleton remodelling, and cellular morphogenesis (Li, Chen et al., 2017).

Recently, there have been two reports on the endocytosis‐related protein Pal1 in *Fusarium graminearum* and *Ustilaginoidea virens* (Chen et al., 2020; Yin et al., 2020). In *F. graminearum*, disruption of *FgPAL1* leads to defects in vegetative growth, conidial morphology, and pathogenicity (Yin et al., 2020). In *U. virens*, in addition to participating in asexual development and full virulence, *UvPal1* also interacts with other endocytic proteins as well as the septin protein Cdc11 (Chen et al., 2020). However, the regulatory mechanism of Pal1 in plant‐pathogenic fungi is still not clear. Interestingly, Pal1 is also predicted to be a mucin protein, so we speculated whether the function of Pal1 related to endocytosis may be similar to MoMsb2, which play roles upstream of both the cAMP and Pmk1 signalling pathways in *M. oryzae*. Consistent with our speculation, in this study we demonstrate that Pal1 affects appressorium formation and virulence through regulating cAMP signalling and the Pmk1–MAPK signalling pathway. A series of biological processes, including utilization of glycogen and lipid, vesicle transport, assembly of actin ring, and localization of septin proteins, were blocked or defective in the *PAL1* deletion mutants. Our study elucidated the mechanism of the endocytosis protein Pal1 regulating appressorium formation and maturation in rice blast fungus.

## 2 RESULTS

### 2.1 Identification of an appressorium highly expressed gene *PAL1* in *M. oryzae*

Through whole transcriptome data (authors’ unpublished data), we found the gene *MGG_02779* was highly expressed at the appressorium development stage of *M. oryzae*. This gene encodes a 526 amino acid protein with a Pal1 domain and eight exons (Figure S1a). We named this gene as *PAL1* in *M. oyzae*. Phylogenetic analysis showed that Pal1 is highly conserved in fungi, and the Pal1 protein of *M. oryzae* has the highest homology with that of *Gaeumannomyces tritici* and *Neurospora crassa*. In addition, *M. oryzae* Pal1 is also closely related to the related proteins of *F. graminearum* and other fungi (Figure S1b).

To confirm the expression pattern and determine the function of *PAL1* in *M. oryzae*, we examined the expression of *PAL1* at different developmental stages of *M. oryzae*. Total RNA extracted from the hyphae (HY), conidia (CO), appressoria at 3 h postinoculation (hpi, AP‐3h) and 12 hpi (AP‐12h), and infectious hyphae at 18 hpi (IH‐18h), 24 hpi (IH‐24h), and 48 hpi (IH‐48h) of *M. oryzae* were used to perform reverse transcription quantitative PCR (RT‐qPCR) analysis. We found the expression level of *PAL1* at AP‐12h was four to five times as much as that of other developmental stages (Figure S2), suggesting that *PAL1* may play an important role in the appressorium of *M. oryzae*.

To determine the function of *PAL1* in *M. oryzae*, we disrupted this gene in the wild‐type strain P131 using a split‐PCR strategy (Goswami, 2012). The transformants were screened by PCR‐based methods (Figure S3a), and the RT‐PCR result indicated that the candidate deletion mutants lacked *PAL1* (Figure S3b). Two independent *PAL1* deletion mutants (PAL1KO1 and PAL1KO2) were obtained and used for the subsequent analyses. The complementary strains were also generated by introducing the 1.5 kb native promoter‐driven *PAL1* coding region into the deletion mutant PAL1KO1. Most of the transformants were recovered in all tested phenotypes, therefore one of the complementation transformants, cPAL1, was used for further experiments.

### 2.2 *PAL1* is involved in endocytosis in *M. oryzae*

**FIGURE 1:**
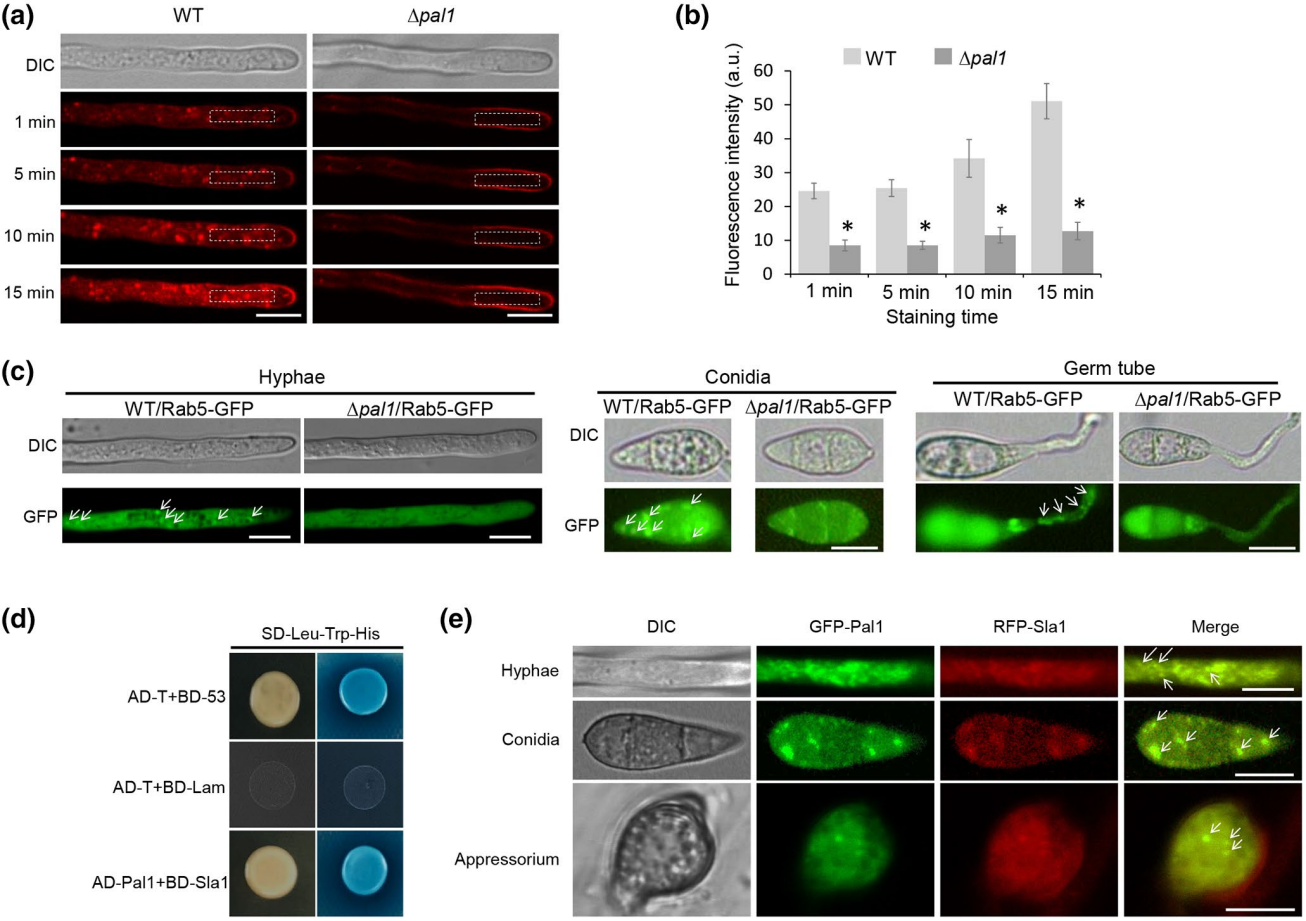
Pal1 plays a role in the endocytosis process. (a) Endocytosis process observation of the hyphal tips at different time points after staining with FM4‐64. Bar = 10 μm. (b) Bar chart showing the fluorescence intensity at the indicated regions of the hyphal tip in (a). At least 10 hyphae of each strain were used for measurement using ImageJ software. Error bars represent standard deviation and asterisks represent significant differences (*p* < 0.01). a.u., arbitrary units. (c) Subcellular localization of the endosome protein Rab5‐GFP in hyphae, conidia, and germ tubes of the wild type and the ∆*pal1* mutant. White arrows indicate the endosomes. Bar = 10 μm. (d) Yeast two‐hybrid (Y2H) assay was performed to detect interaction between Pal1 and Sla1. The blue interaction between pGBKT7‐53 (BD‐53) and pGADT7‐T (AD‐T) was used as the positive control, and interaction between pGBKT7‐Lam (BD‐Lam) and pGADT7‐T was used as the negative control. Positive colonies on SD/−Leu−Trp−His were stained with X‐α‐gal. (e) Colocalization of Pal1 and Sla1. White arrows indicate the puncta. Bar = 10 μm

Pal1 was first reported to be gathered in the initial stage of endocytosis in budding yeast (Carroll et al., 2012). We tested whether Pal1 plays similar roles in the rice blast fungus. In the process of cell active transport, the dye FM4‐64 can enter cells and specifically bind to vesicle membranes and other structures (Penalva, 2005). To examine whether Pal1 is required for endocytosis, we used FM4‐64 to stain the hyphae of different strains, and then observed the endocytosis process. The FM4‐64 dye quickly appeared in the cytoplasm at the hyphal tip of the wild‐type strain P131 but stayed on the cytoplasmic membrane of the Δ*pal1* mutant after 1 min of staining. Five minutes later, stronger fluorescence signal showed in the cytoplasm of the wild type stained with FM4‐64 dye, which still showed in the plasma membrane of the Δ*pal1* mutant. After 15 min of staining, most wild‐type mycelia were stained internally but there was still only weak internal fluorescence in the cytoplasm of the Δ*pal1* mutant (Figure 1a,b). This experiment indicated that Pal1 is indeed involved in endocytosis in *M. oryzae*.

To further determine whether Pal1 is required for endosome‐mediated signal compartmentalization and transduction, we fused Rab5, an endosome marker protein, with green fluorescent protein (GFP). The Rab5‐GFP construct was transformed into the wild‐type and Δ*pal1* mutant strains. When the localization of Rab5‐GFP in the hyphal tips was observed, we found that bright puncta were distributed in the cytoplasm adjacent to the cell membrane in the wild type but not in the Δ*pal1* mutant (Figure 1c). A similar phenomenon was also clearly observed in the conidia and germ tubes (Figure 1c). This suggests that endosomal trafficking is involved in Pal1‐mediated endocytosis.

### 2.3 Pal1 interacts and colocalizes with the endocytic protein Sla1

Previous studies reported that in yeast, Pal1p reaches the endocytosis site at the earliest stage of the endocytosis process, then works together with other proteins such AP2 complex proteins, Sla1p, and Ent1p (Carroll et al., 2012). Therefore, we conducted yeast two‐hybrid (Y2H) assay to test whether Pal1 interacted with those proteins present at the earliest stage of endocytosis in *M. oryzae*. As expected, the Y2H experiment showed that Pal1 can interact with Sla1 in *M. oryzae* (Figure 1d).

To further determine whether Pal1 is colocalized with Sla1 in *M. oryzae*, we generated GFP‐Pal1 and red fluorescent protein (RFP)‐Sla1 constructs driven by the *PAL1* and the *SLA1* native promoters, respectively. The GFP‐Pal1 fusion vector was transformed into the Δ*pal1* mutant strain. Phenotypes of the positive transformants, including vegetative growth, conidiation, and virulence, were all recovered to the wild type state (data not shown), indicating that the GFP‐Pal1 construct functioned fully. Subsequently, the GFP‐Pal1 and RFP‐Sla1 constructs were transformed into the wild‐type strain. Green and red fluorescence signals were both observed in the cytoplasm with some puncta in the hyphae, conidia, and appressoria (Figure S4). To further confirm the relationship of localization of the two proteins, we cotransformed the GFP‐Pal1 and RFP‐Sla1 constructs into the Δ*pal1* mutant to observe their localization at different developmental stages. The results showed that Pal1 and Sla1 colocalized in the cytoplasm as well as in puncta structures in all tested samples, including the mycelium, conidium, and appressorium (Figure 1e). Thus, we concluded that Pal1 interacted and was colocalized with the early‐stage endocytic protein Sla1 in *M. oryzae*, suggesting that it also plays a key role in the initial stage of endocytosis.

### 2.4 Pal1 is important for cell wall integrity

**FIGURE 2:**
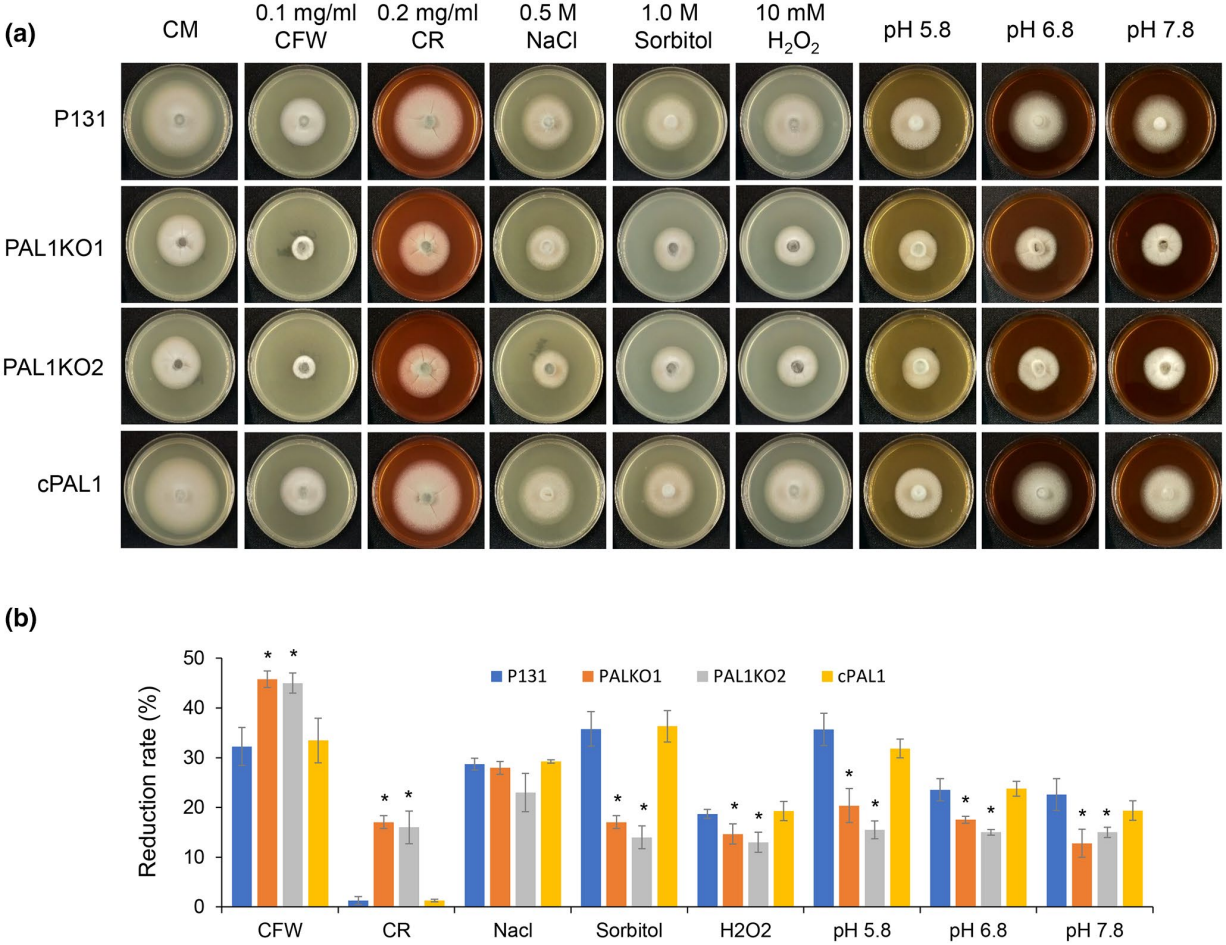
*PAL1* affects various stress responses. (a) Colony of different strains on complete medium (CM) plates supplemented with different stress reagents or pH buffers. (b) Growth reduction rates of different strains on various stress reagents. For each strain, the reduction rate was calculated by comparing the colony diameter on treatments with that of CM without treatment. Significant differences are marked with a single asterisk (*p* < 0.05)

We then tested whether Pal1 is involved in resistance to environmental stress during fungal development. Various stresses, including cell wall inhibitors (0.1 mg/ml Calcofluor white [CFW], 0.2 mg/ml Congo red [CR]), high osmotic pressure (0.5 M NaCl, 1 M sorbitol), and oxidative stress (H_2_O_2_) were added into a complete medium (CM) plate for testing. Acidic or alkaline pH was also used for testing, by adjusting the pH of the CM medium to different values (pH 5.8, 6.8, and 7.8). Compared with the wild‐type and complementary strains, the Δ*pal1* mutant was more sensitive to CFW and CR, indicating that *PAL1* is involved in cell wall integrity. On the other hand, the Δ*pal1* mutant was more resistant to 1 M sorbitol, 10 mM H_2_O_2_, and acidic/alkaline pH conditions (Figure 2a,b), suggesting that deletion of *PAL1* may lead to compensation effects in the fungal response to these stresses. Together, our results show that deletion of *PAL1* affects the fungal response to different stresses.

### 2.5 *PAL1* regulates asexual growth and conidial formation of *M. oryzae*

**FIGURE 3:**
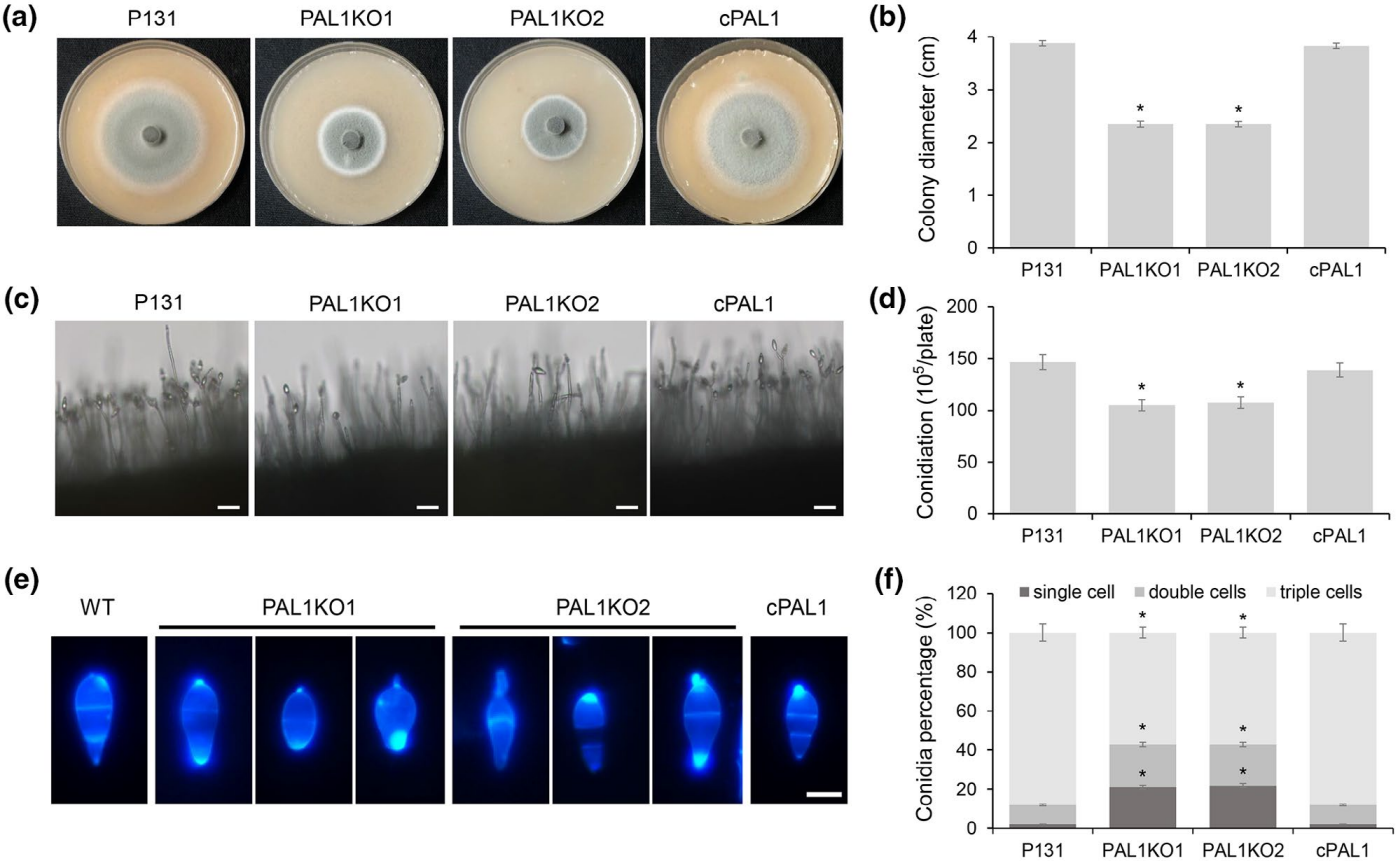
*PAL1* is important for asexual development of *Magnaporthe oryzae*. (a) Colony of the wild type (P131, WT), PAL1 deletion mutants (PAL1KO1 and PAL1KO2), and the complementary strain (cPAL1) on oatmeal tomato agar (OTA) plates. (b) Colony sizes of different strains. Significant differences are labelled with single asterisks (*p* < 0.05). (c) Conidiophore morphology formed by different strains grown on OTA plates. Bar = 50 μm. (d) Conidiation of different strains on OTA plates. Significant differences are labelled with single asterisks (*p* < 0.05). (e) Conidial morphology of different strains stained by Calcofluor white. Bar = 10 μm. (f) Statistical analysis of conidia with different cell numbers produced by different strains. Significant differences are labelled with single asterisks (*p* < 0.05)

To investigate the role of *PAL1* in fungal growth and development, we cultured the Δ*pal1* mutants, the wild type, and the complementary strain on oatmeal tomato agar (OTA) medium for 5 days at 28°C. The colony diameters of the Δ*pal1* mutants were significantly smaller than those of the wild type and complementary strains (Figure 3a,b). The CFW staining assay showed the mycelial cell length of the mutants was obviously shorter than that of the wild type (Figure S5). This result accounts for the decreased growth rate of the mutants compared with that of the wild type. Therefore, *PAL1* plays an important role in fungal vegetative growth.

We also examined the effect of *PAL1* deletion on spore formation and development. Although the deletion mutant could form normal conidia, the number of spores attached to conidia was significantly less than that of wild‐type and complementary strains (Figure 3c). In comparison, the number of conidia of the two deletion mutants was 36% less than that of the wild‐type and complementary strains (Figure 3d). This result is consistent with the observation of conidiophores, and demonstrates that *PAL1* affects spore formation of *M. oryzae*. Moreover, the spore morphology of Δ*pal1* was evidently different from that of the wild type. Around 60% of Δ*pal1* conidia did not show the normal two/septa/three‐cells morphology. By contrast, 90% of P131 and cPAL1 spores formed two‐septa conidia, and only 10% of their spores formed one or no septum (Figure 3e,f). These results suggest that *PAL1* is involved in maintaining the spore morphology of *M. oryzae*.

### 2.6 *PAL1* is responsible for full virulence of *M. oryzae*

**FIGURE 4:**
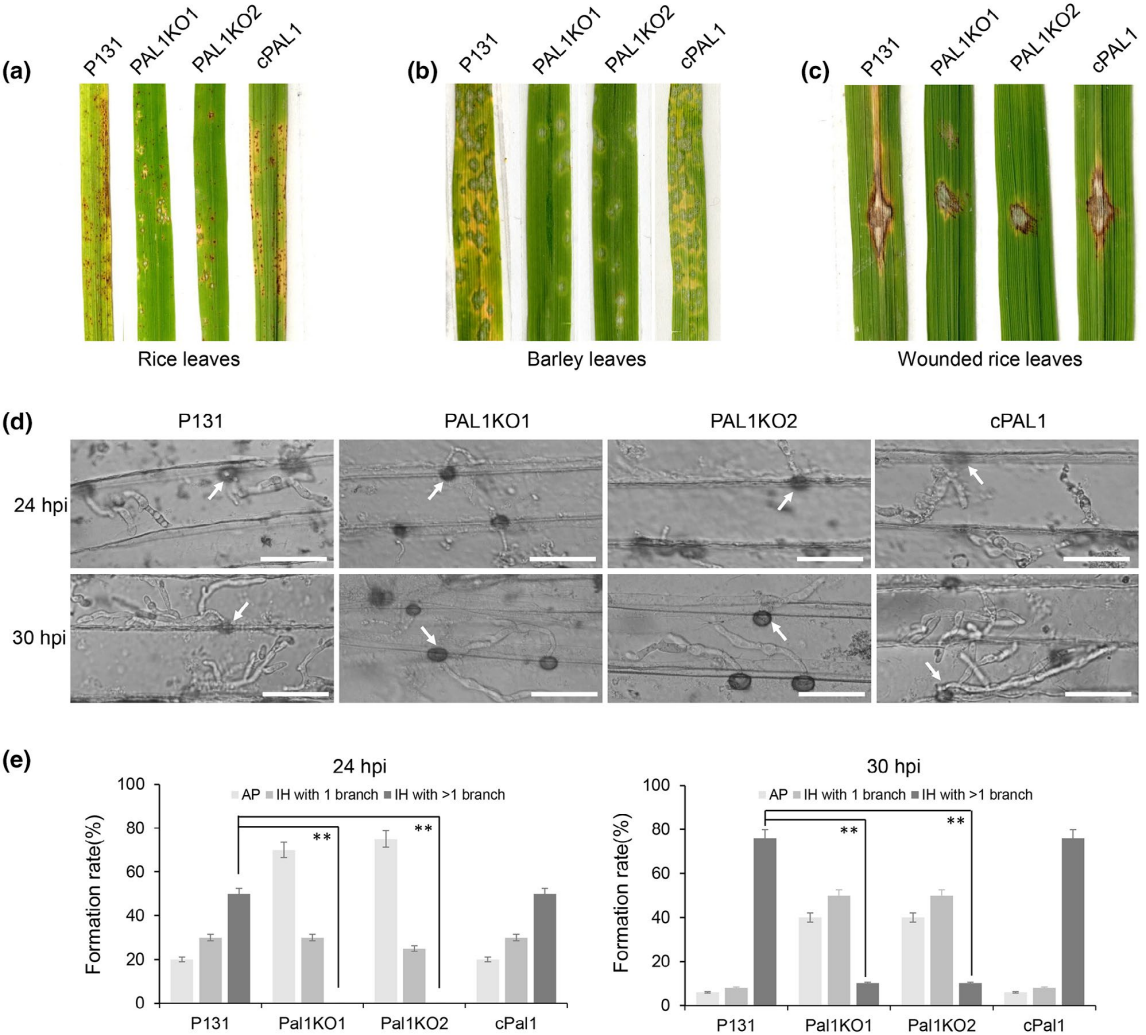
*PAL1* is responsible for full virulence and invasive growth in host cells. Rice leaves (a) and barley leaves (b) were inoculated with conidial suspension of wild type (P131), PAL1 deletion mutants (PAL1KO1 and PAL1KO2), and the complementary strain (cPAL1). Typical leaves were photographed at 5 days postinoculation (dpi). (c) Virulence test on wounded rice leaves. Rice leaves were slightly scratched by a needle followed by inoculation with a mycelial block of different strains. (d) Penetration and infectious hyphae of P131, PAL1KO1, PAL1KO2, and cPAL1 were examined at 24 and 30 h postinoculation (hpi). Arrows indicate appressoria. Bar = 50 μm. (e) Percentages of appressoria (AP) with different types of infectious hyphae (IH) at 24 and 30 hpi. Asterisks indicate statistically significant differences (*p* < 0.01)

To detect the effect of *PAL1* deletion on the pathogenicity of *M. oryzae*, we sprayed rice and barley leaves with a conidial suspension of Δ*pal1*, P131, and cPAL1. After 4 days, only a small number of disease spots formed on the plant leaves inoculated by Δ*pal1*. However, more and bigger lesions appeared on the leaves inoculated with P131 and cPAL1 (Figure 4a,b). These results indicate that *PAL1* plays an important role in the full virulence of *M. oryzae*.

To determine whether the expansion of fungal growth in Δ*pal1* was affected, rice seedlings were scratched with needles, and mycelial blocks of different strains were inoculated to the inoculation site. Four days later, we observed that the leaves of rice inoculated with P131 and cPAL1 showed extended disease lesions, while lesions of leaves inoculated with Δ*pal1* could not extend beyond the inoculation sites (Figure 4c). This evidence suggests that the expansion of Δ*pal1* in host cells is blocked.

To further confirm if the expansion of the infectious hyphae was blocked in Δ*pal1*, the formation of invasive hyphae was observed in barley epidermis cells. Statistical analysis showed that at 24 hpi more than 80% of P131 or cPAL1 attachment cells formed infectious hyphae with one or more branches, while about 70% of Δ*pal1* conidia could only form appressoria (Figure 4d,e). At 30 hpi, more than 70% of appressoria of P131 or cPAL1 had formed expanded hyphae, while only 10% of appressoria of Δ*pal1* formed secondary infectious hyphae and nearly 40% of fungal cells still stayed at the appressorium stage (Figure 4d,e). Obviously, the infection rate of Δ*pal1* was significantly lower than that of the wild‐type and complementary strains. These results indicate that *PAL1* is responsible for both appressorium‐mediated penetration and infectious hyphae expansion.

### 2.7 Pal1 is involved in the inhibiting host defence response

On fungal infection, plant cells often produce reactive oxygen species (ROS) for resistance. Because Δ*pal1* was deficient in colonization in host cells, we detected ROS accumulation in host cells infected with the *PAL1* deletion mutant. The 3,3′‐diaminobenzidine (DAB) staining assay was conducted on barley epidermis inoculated with a conidial suspension of Δ*pal1*, P131, and cPAL1 at 30 hpi. We observed and photographed the epidermal cells under the microscope after decolourization. More than 60% of the plant cells inoculated with Δ*pal1* showed a reddish brown colour, representing massive ROS accumulation, while less than 20% of the cells infected by P131 or cPAL1 were stained, suggesting less ROS accumulation (Figure S6a,b). These data indicate more ROS accumulated in plant cells infected by Δ*pal1* than by P131 or cPAL1, which suggests that *PAL1* is involved in inhibiting host defence response.

### 2.8 Pal1 regulates appressorium formation

**FIGURE 5:**
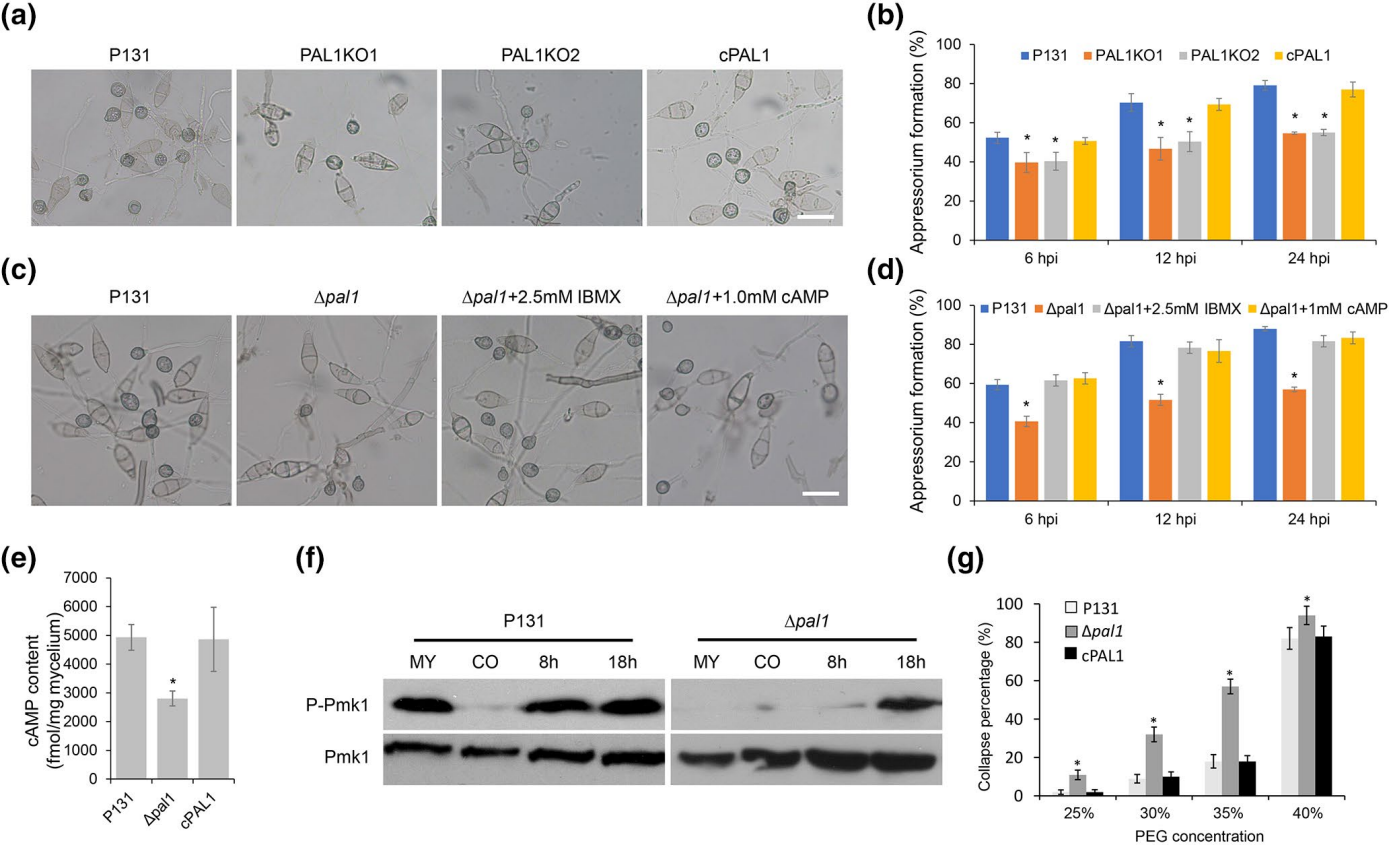
Pal1 regulates the cAMP and Pmk1–MAPK signalling pathways for appressorium formation. (a) Appressorium formation of different strains on hydrophobic surfaces. Bar = 25 μm. (b) Statistical analyses of appressoria formation rate at different time points in (a). Asterisks indicate statistically significant differences (*p* < 0.05). (c) Appressoria formation of different strains on hydrophobic surfaces supplemented with 2.5 mM 3‐isobutyl‐1‐methylxanthine (IBMX) and 1 mM cAMP. Bar = 25 μm. (d) Statistical analyses of appressoria formation rate at different time points in (c). Asterisks indicate statistically significant differences (*p* < 0.05). (e) Quantification of intracellular cAMP levels. The intracellular cAMP levels were detected in the mycelial stage. Asterisks indicate statistically significant differences (*p* < 0.05). (f) Pmk1 phosphorylation level analysis. Total proteins were extracted from different samples and the phosphorylation levels of Pmk1 were detected by a phospho‐p44/42 MAPK antibody. The endogenous Pmk1 was detected by a p44/42 MAPK antibody. (g) Cytorrhysis assay. Drops of conidial suspension (10^5^ conidia/ml) were placed on the hydrophobic surface and treated with different concentrations of polyethylene glycol (PEG) 8000. Asterisks indicate statistically significant differences (*p* < 0.05)

Considering that Pal1 is highly expressed in appressoria, we wondered whether deletion of *PAL1* might affect appressorium formation. Conidia of different strains were inoculated on hydrophobic glass slides and were used to observe appressorium formation at different time points (6, 12, and 24 h). At 6 hpi, many immature appressoria formed at the tip of the germ tube in Δ*pal1*, while half of the wild‐type conidia had formed mature appressoria. At 12 hpi, around 70% of P131 or cPAL1 conidia produced mature appressoria, much higher than in the deletion mutant. At 24 hpi, more than 80% of the wild‐type conidia formed mature appressoria, while it was only about 50% in Δ*pal1* (Figure 5a,b). In summary, the appressorium formation rate of Δ*pal1* was markedly slower than that of the wild‐type and complementary strains, indicating that Pal1 regulates appressorium formation in *M. oryzae*.

### 2.9 Pal1 plays a role upstream of the cAMP signalling pathway

In *M. oryzae*, the cAMP signalling pathway is one of the main pathways regulating appressorium formation. The cellular cAMP concentration is closely determined by adenylate cyclase and phosphodiesterase, which are responsible for synthesis and degradation of cAMP, respectively (Sassone‐Corsi, 2012). Due to the defect of Δ*pal1* in appressorium formation, we speculated whether Pal1 acts upstream of the cAMP signalling pathway. We exogenously added a membrane‐permeable cAMP variant, 8‐Na‐cAMP, or a cyclophosphodiesterase inhibitor, 3‐isobutyl‐1‐methylxanthine (IBMX), to increase the cAMP level during the formation of appressoria. At 6 hpi, the percentage of appressorium formation of Δ*pal1* was only 40% in water. When the Δ*pal1* conidial suspension was supplemented with 1 mM cAMP or 2.5 mM IBMX, the percentage of appressorium formation was restored to around 60%, comparable to that of the wild type. At 12 and 24 hpi, adding cAMP or IBMX increased the appressorium formation ratio of Δ*pal1* to the wild‐type level (Figure 5c,d). These results indicate that Pal1 plays a role upstream of the cAMP signalling pathway in *M. oryzae*. Consistent with this, we found that the content of endogenous cAMP in the mycelium of Δ*pal1* was reduced nearly twice as much as that of the wild‐type strain (Figure 5e). This suggests that Pal1 plays an important role in cAMP signalling pathway‐mediated appressorium formation in *M. oryzae*.

### 2.10 Pal1 is require for Pmk1 phosphorylation

The observation of the infection process showed that Pal1 is also important for appressorium‐mediated penetration. Considering that the Pmk1 signalling pathway is required for appressorium maturation and penetration, we speculated whether Pal1 also regulates the Pmk1 signalling pathway. We detected the phosphorylation level of the Pmk1 protein in different strains. Proteins of different stages of the wild type and Δ*pal1* mutant were extracted and analysed by western blot. No visible difference was found in the protein level of Pmk1 (42 kDa) between wild type (P131) and Δ*pal1* (Figure 5f, bottom panel). However, when using the phosphor‐MAPK antibody for testing, Pmk1 phosphorylation was clearly detected at the mycelium, 8 h appressorium, and 18 h appressorium stages, while only a weak signal was detected in the conidium. In contrast, reduced and delayed Pmk1 phosphorylation were detected in the Δ*pal1* appressoria (Figure 5f, upper panel). This finding suggests that Pal1 also contributes to Pmk1 phosphorylation and plays a role upstream of the Pmk1 signalling pathway during appressorium development.

### 2.11 Pal1 affects utilization of glycogen and lipid during appressorium formation

**FIGURE 6:**
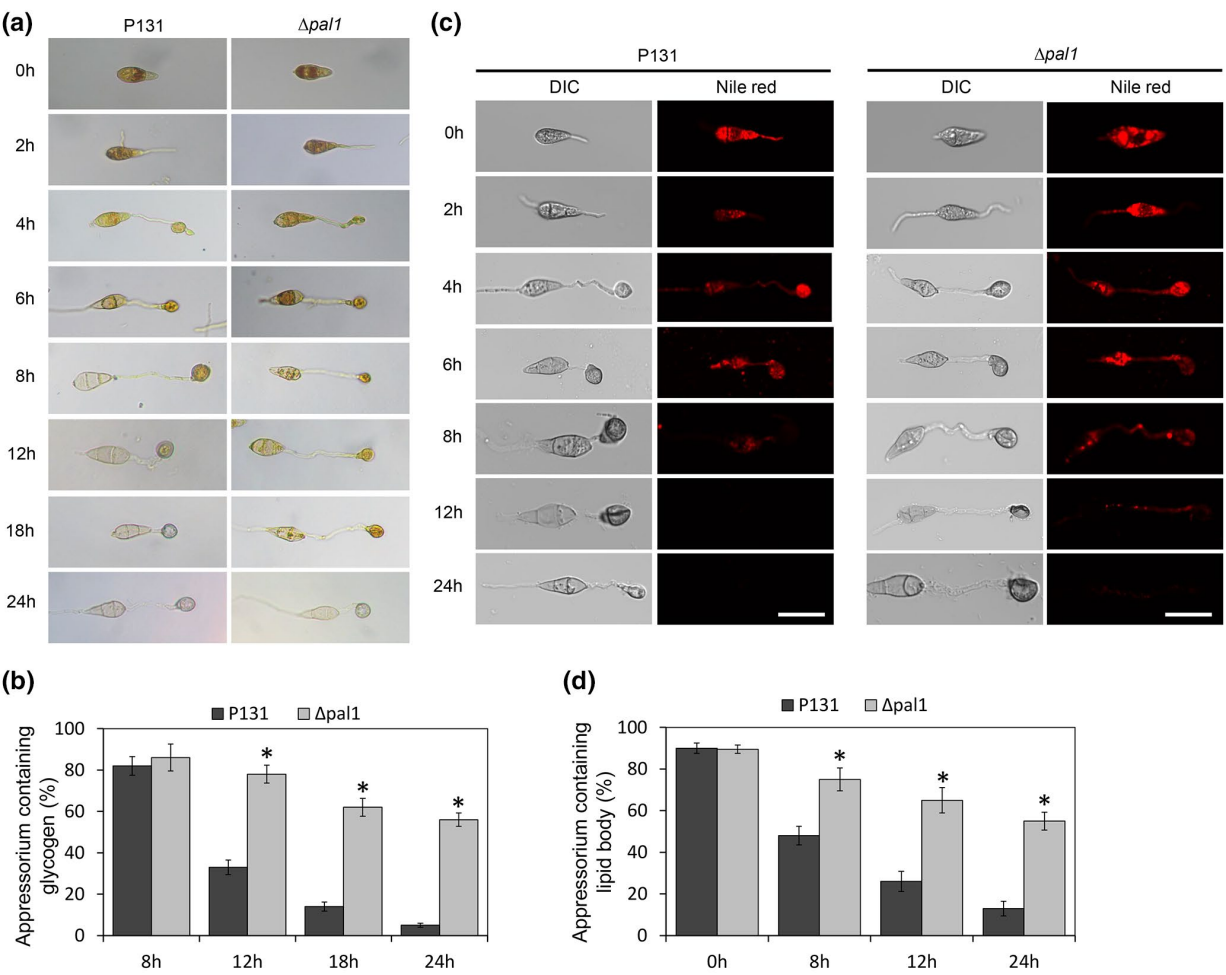
Pal1 affects utilization of glycogen and lipids in appressoria. (a) Glycogen in conidia and appressoria were stained with KI/I_2_ and observed at different time points. Bar = 25 μm. (b) Quantitation of the total glycogen content in appressoria in the indicated strains. Means and standard errors were calculated from three independent replicates. Asterisks indicate statistically significant differences (*p* < 0.05). (c) Lipids in conidia and appressoria were stained with Nile red and observed at different time points. Bar = 25 μm. (d) Quantitative analysis of lipid bodies during appressorium formation in the indicated strains. Means and standard errors were calculated from three independent replicates. Asterisks indicate statistically significant differences (*p* < 0.05)

Glycogen and lipids stored in conidia are nutrients required for appressorium formation and maturation in *M. oryzae*. Utilization of glycogen and lipids directly determines the function of the appressorium. Because Pal1 plays roles upstream of both the cAMP and Pmk1 signalling pathways, we inferred that utilization of glycogen and lipid should be blocked in the Δ*pal1* mutant. Different time points during appressorium formation of the wild type and Δ*pal1* were stained with I_2_/KI to detect glycogen utilization and with Nile red to detect lipid utilization. The glycogen staining assay showed that in the wild‐type strain, glycogen began to transfer from spore to appressorium at 8 h and was totally utilized at 12 h. In the Δ*pal1* mutant, glycogen could still be observed by staining at 18–24 h (Figure 6a), indicating that the utilization of glycogen in Δ*pal1* was severely blocked. Similar results were found in lipid utilization. In the wild type, lipid droplets began to be degraded at 8 h, and could not be detected at 12 h. In Δ*pal1*, lipid droplets could be still detected at 12 h (Figure 6b), indicating that the utilization of lipid in Δ*pal1* was delayed. These results suggest that Pal1 affects the metabolism of glycogen and lipid during transition from the conidium to appressorium.

### 2.12 Pal1 is important for autophagy

**FIGURE 7:**
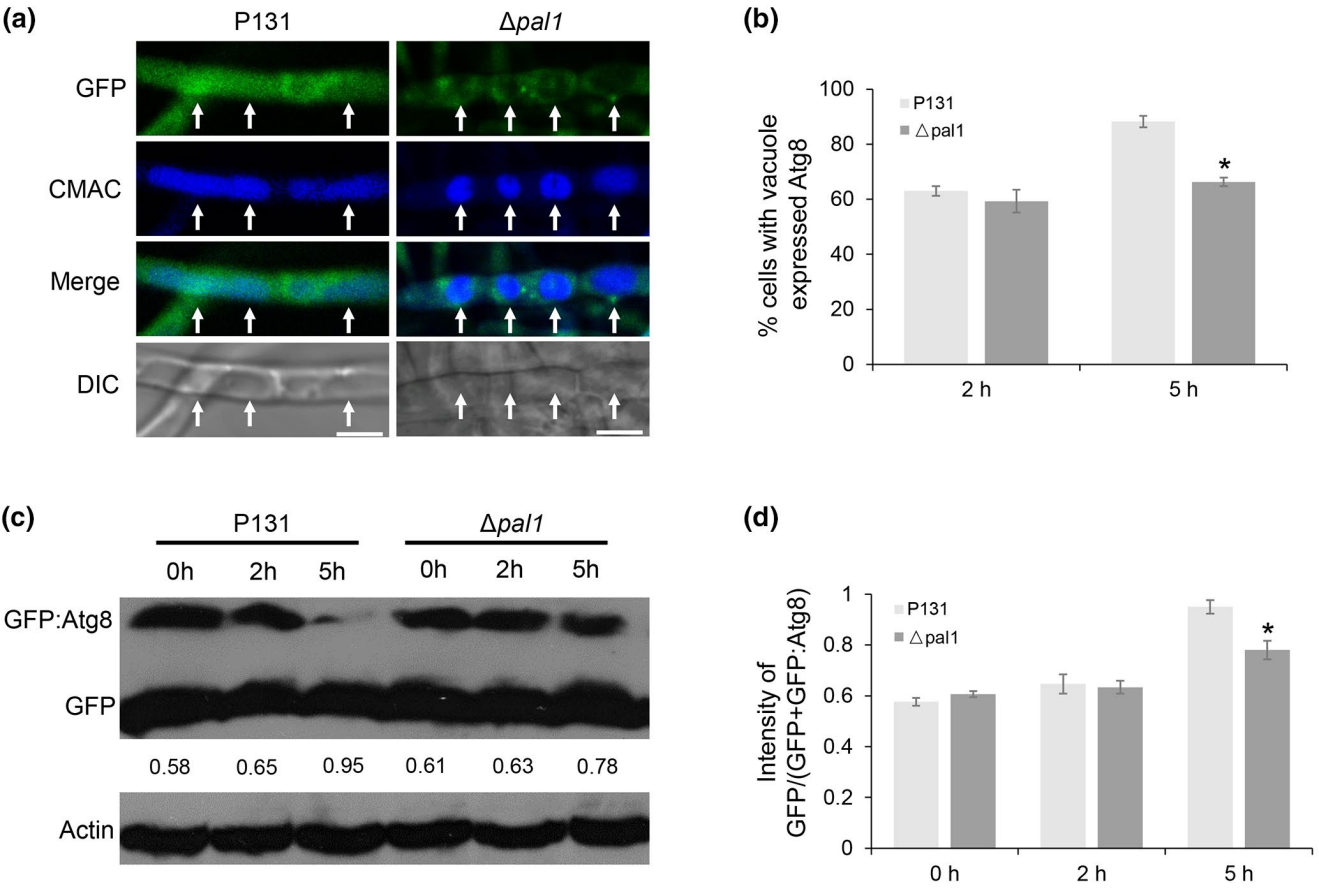
Pal1 is important for autophagy. (a) Hyphae of the wild type (P131) and ∆*pal1* expressing green fluorescent protein (GFP):Atg8 and stained with 7‐amino‐4‐chloromethylcoumarin (CMAC) were exposed to nitrogen starvation for 5 h. Arrows indicate the vacuoles. Bar = 5 μm. (b) Statistic analysis of mycelial cells with vacuoles expressing GFP:Atg8. Significant differences are marked with single asterisks (*p* < 0.05). (c) Expression levels of GFP:Atg8 and free GFP after nitrogen starvation were detected by western blot with anti‐GFP antibody. Protein levels were normalized using an anti‐actin antibody. (d) Statistic analysis of signal intensity ratio of GFP/(GFP+GFP:Atg8) after nitrogen starvation. Significant differences are marked with single asterisks (*p* < 0.05)

Nutrition transition from the conidia to appressoria is a consequence of autophagy followed by mitosis and nuclear migration (Veneault‐Fourrey et al., 2006). Because Pal1 influences the transition of glycogen and lipid, we determined whether or not disruption of *PAL1* affects the autophagy process. Mycelia of the wild‐type and Δ*pal1* strains were cultured in liquid minimal medium (MM) with reduced nitrogen (MM−N). We used *GFP:MoATG8* as a functional marker to detect delivery of vesicles to vacuoles and the breakdown of autophagosomes. Free GFP cannot be hydrolysed from GFP:MoAtg8 in normal autophagy (Kim et al., 2001; Mizushima et al., 2004). Therefore, we examined autophagy by detecting GFP:MoAtg8 in mycelia under nitrogen starvation. A GFP signal was detected in 63% of hyphal cells of wild type (P131) and 59% of cells of Δ*pal1* after 2 h of nitrogen starvation treatment. However, after 5 h of nitrogen starvation, a GFP signal was observed in 88% of wild type mycelial cells, significantly higher than the 68% in Δ*pal1* cells. This phenomenon was confirmed by a staining assay with the vacuole dye 7‐amino‐4‐chloromethylcoumarin (CMAC) (Figure 7a,b). Furthermore, western blotting was conducted to detect proteolysis of GFP:MoAtg8. Total proteins were extracted from wild type and Δ*pal1* expressing GFP:MoAtg8 followed by 0, 2, and 5 h of culture in MM−N medium. Free GFP and the fusion protein GFP:MoAtg8 (41 kDa) were then detected using the anti‐GFP antibody. The ratio of GFP intensity to the sum of GFP:MoAtg8 and GFP (GFP+GFP:MoAtg8) were measured. In normal conditions and MM−N exposure for 2 h, the ratio was comparable in wild type and Δ*pal1*. After 5 h of nitrogen starvation, the ratio increased to 0.95 in wild type, significantly higher than the 0.78 in Δ*pal1* (Figure 7c,d), indicating autophagy was blocked in Δ*pal1*. Based on this, we conclude that disruption of *PAL1* influences autophagy.

### 2.13 Turgor accumulation is reduced in the Δ*pal1* mutant

Because the utilization of glycogen and lipid droplets was severely reduced in the Δ*pal1* mutant, and the autophagy process was also blocked in the mutant, we inferred that the appressorial turgor should be also reduced in the mutant. Therefore, turgor accumulation was examined in appressoria of the Δ*pal1* mutant by a cytorrhysis assay. When treated with different concentrations of polyethylene glycol (PEG) 8000, the mature appressoria formed by the Δ*pal1* mutant were more easily collapsed than those of the wild type (Figure 5g), suggesting the turgor pressure in the Δ*pal1* mutant is significantly reduced.

### 2.14 Pal1 regulates the assembly of actin rings in appressorium

**FIGURE 8:**
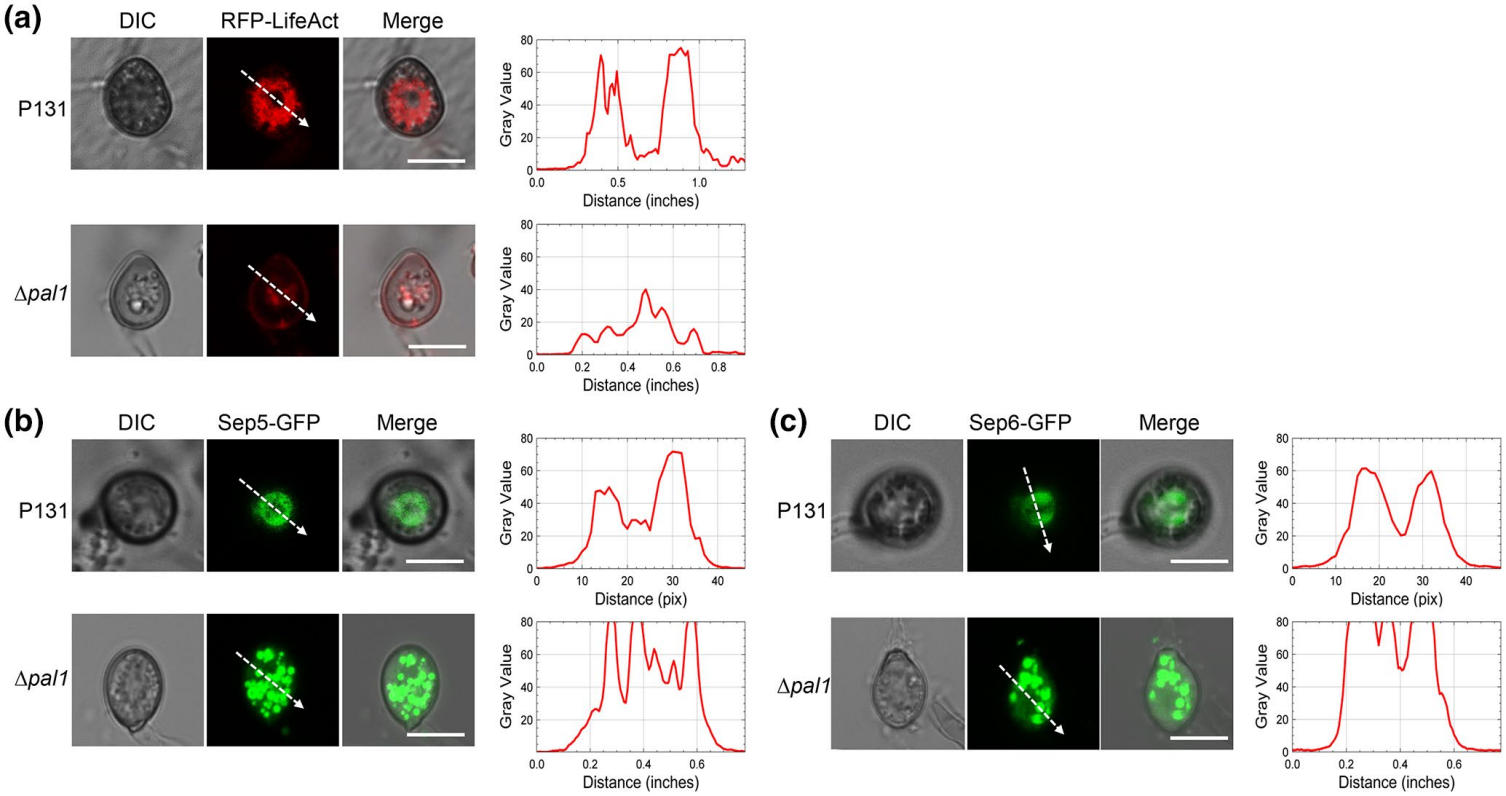
Pal1 regulates actin ring assembly and localization of septin proteins. (a) Observation of the actin ring in appressoria of wild type (P131) and ∆*pal1* expressing RFP‐Lifeact. (b) Localization of Sep5‐GFP in wild type and ∆*Pal1*. Bars = 10 μm. (c) Localization of Sep6‐GFP in wild type and ∆*Pal1*. Bars = 10 μm. For (a) to (c), linescan graphs show fluorescence intensity in a transverse section of individual appressoria as the arrow‐indicated direction

During the development of appressorium, the actin ring, which is located at the base of the infected cell surrounding the appressorium pore, provides cortical rigidity and promotes the emergence of the penetration peg to invade the host plant. Because Δ*pal1* was defective in appressorium maturation and plant infection, we examined whether the deletion of *PAL1* affected actin ring formation in the appressorium. The F‐actin marker Lifeact‐RFP was transformed into the wild‐type and Δ*pal1* mutant strains. At 24 hpi, 85% of wild‐type appressoria formed an actin ring, while 70% of Δ*pal1* appressoria exhibited dispersed actin localization that failed to form a ring (Figure 8a). This result suggests that Pal1 is required for assembly of the actin ring in the appressorium.

### 2.15 Pal1 determines localization of septin proteins

The actin ring is organized with the assistance of septin proteins (Dagdas et al., 2012). We investigated whether localization of septin proteins was affected by Pal1. GFP‐Sep5 and GFP‐Sep6 constructs were transformed into the wild‐type strain or the Δ*pal1* mutant. Positive transformants were used to observe the localization of GFP‐Sep5 and GFP‐Sep6. In the wild‐type strain, GFP signals gathered as a ring in the centre of the appressorium, while in the Δ*pal1* mutant, GFP‐Sep5 and GFP‐Sep6 were mainly distributed in granular form throughout the appressorium and did not form a ring (Figure 8b,c). These results indicate that Pal1 is responsible for accurate localization of septins.

## 3 DISCUSSION

As previously reported, Pal1p is very important for maintaining the cylindrical cell morphology in *Schizosaccharomyces pombe* (Ge et al., 2005) and *S. cerevisiae* (Carroll et al., 2012). Deletion of *S. cerevisiae* Pal1p results in spherical or pear‐shaped cell morphology (Carroll et al., 2012). Recently, two studies in the plant‐pathogenic fungi *F. graminearum* and *U. virens* have demonstrated roles for Pal1 in vegetative growth, conidial morphology, pathogenicity, polarized tip growth, and developments of ascospores (Chen et al., 2020; Yin et al., 2020). However, the Pal1 regulatory mechanisms in plant‐pathogenic fungi are still not well understood. In this study, we revealed that Pal1‐mediated endocytosis plays key roles in appressorium formation and maturation, which function upstream of both cAMP and Pmk1–MAPK signalling pathways in *M. oryzae*.

For many filamentous fungi, the appressorium is a specialized structure required to penetrate host tissue for colonization. The expression level of *PAL1* in appressoria was obviously higher than that in vegetative hyphae, conidia, and infectious hyphae. We speculated that Pal1 may play a key role during appressorium formation and maturation. Several important signalling pathways, including the cAMP signalling pathway (Lee & Dean, 1993; Xu & Hamer, 1996), the Pmk1–MAPK signalling pathway (Xu & Hamer, 1996; Zhao et al., 2005), the Ca^2+^ signalling pathway (Lee & Lee, 1998), and the TOR signalling pathway (Marroquin‐Guzman & Wilson, 2015; Marroquin‐Guzman et al., 2017), are involved in regulating the formation and maturation of appressoria. In this study, we detected that the content of cAMP was less in Δ*pal1* than in the wild type. Exogenous addition of cAMP content restored the appressorium formation ratio of Δ*pal1* to the wild‐type level. This evidence suggests that *PAL1* affects appressorium formation by regulating the cAMP signalling pathway. We also found the Pmk1 phosphorylation level was significantly reduced in the Δ*pal1* mutant, suggesting that Pal1 may regulate the Pmk1–MAPK signalling pathway. All these suggest Pal1is a key upstream signalling regulator for appressorium formation.

MoMsb2 is a mucin protein responsible for cell surface recognition. The phosphorylation level of Pmk1 was reduced in *MoMSB2* deletion mutants, indicating that MoMsb2 may function upstream of the Pmk1 MAPK cascade (Liu et al., 2011). The G protein coupled receptor (GPCR) Pth11 is a receptor protein involved in surface sensing. Disruption of *PTH11* leads to defects in appressorium formation, which can be recovered by exogenous cAMP (DeZwaan et al., 1999). *MSB2* and *PTH11* may be functionally related in sensing signals of the leaf surface, but the detailed mechanism is still unknown. Interestingly, Pal1 is also a predicted mucin protein, suggesting endocytosis could play a role in surface signal transduction. Whether or not MoMsb2 is also involved in endocytosis is fascinating. Previous study for MoEnd3, another endocytic protein, indicated that MoEnd3 mediates the transport of Pth11 (DeZwaan et al., 1999; Li et al., 2010) and membrane sensor MoSho1, triggers the downstream phosphorylation of Pmk1, and promotes autophagy, appressorium formation, and penetration (Li, Gao et al., 2017). Deletion of *MoEND3* delays the endocytosis of Pth11 and MoSho1 and affects appressorium formation, which proves that endocytosis plays an important role in the signal transduction and pathogenesis of *M. oryzae* (Li, Gao et al., 2017). Together with these studies, our study shows that Pal1‐mediated endocytosis is closely connected with cAMP and Pmk1 signalling pathways by transducing upstream signals to downstream signalling pathways for appressorium formation and maturation. However, considering that Pal1 is an endocytosis factor, it could also be possible that Pal1 affected all the prerequisite processes required for appressorium formation, not only required for appressorium formation, by acting upstream of important signalling pathways.

The actin ring is a ring structure of the hetero‐oligomer from mature appressorium, supporting enough mechanical pressure to form the penetration peg to infect the plant epidermis. During the development of the appressorium, an actin ring will form in the centre of the cell to assist penetration peg formation (Dagdas et al., 2012). The septin proteins Sep3, Sep4, Sep5, and Sep6 are involved in the assembly of the actin ring and support F‐actin to form cytoskeleton in the appressorium (Dagdas et al., 2012). In this study, using the actin marker Lifeact‐RFP, we found standard actin ring formation was impaired in Δ*pal1* by affecting localization of the septins. This suggests Pal1 is involved in the assembly of the actin ring for appressorial penetration. Interestingly, in *U. virens*, Pal1 was also proved to interact with a septin protein Cdc11 (Chen et al., 2020), suggesting that this regulatory mechanism may be conserved in fungi.

In yeast, another Pal protein, Pal2, is also known to participate in clathrin‐mediated endocytosis. Double deletion of *PAL1* and *PAL2* causes a slightly slower growth defect than the wild type (Ghosh et al., 2021), while in *M. oryzae* no Pal2 homologous protein was found. However, one protein (MGG_00684) with low similarity to yeast Pal2 was found and also annotated as a Pal1‐like protein. As many proteins are involved in endocytosis, the viability of the strain lacking *PAL1* in *M. oryzae* may be a result of functional redundancy of Pal1 and Pal2, as well as other unknown endocytic proteins that remain to be discovered.

Altogether, Pal1 regulates cAMP and Pmk1 signalling pathways, and as a consequence many important processes in appressorium differentiation and maturation are regulated by Pal1, including glycogen and lipid droplet utilization, appressorial autophagic process, actin ring formation, and turgor accumulation. Considering that Pal1 is very important for functional appressorium formation, and it is well‐conserved and specific in fungi, this membrane‐associated protein may be a valid target for developing broad‐spectrum fungicides.

## 4 EXPERIMENTAL PROCEDURES

### 4.1 Strains and culture conditions

P131 was used as the wild‐type strain. All strains listed in Table S1 were cultured on OTA plates at 28°C. For genomic DNA and RNA extraction, all strains were cultured in liquid CM at 28°C. Colony diameters on the OTA plates were measured at 120 hpi. Conidia from 7‐day‐old colonies cultured on OTA plates were harvested for experiments.

To test fungal response to environmental stresses, strains were inoculated onto the CM plates supplemented with 0.2 mg/ml CR (Sigma‐Aldrich), 0.1 mg/ml CFW (Sigma‐Aldrich), 0.5 M NaCl, 1 M sorbitol, and 10 mM H_2_O_2_. The colony diameters were measured after 5 days postinoculation (dpi).

### 4.2 RT‐qPCR

To evaluate the *PAL1* expression profile, tissues harvested from different developmental stages (Liu et al., 2018) were used to extract total RNA for preparing the cDNA templates. The qPCR was performed by using an SYBR Green PCR Master Mix kit (Takara) on an ABI 7500 real‐time PCR system (Applied Biosystems).

### 4.3 Gene disruption and complementation

A split‐PCR strategy was used for gene disruption as previously described (Goswami, 2012). Left boarder and right boarder were confirmed by PCR using the Pal1LBCK/ HYG‐LBCK and Pal1RBCK/HYG‐RBCK primer pairs. Gene deletion was further confirmed by RT‐PCR of a c.900 bp internal fragment of *PAL1*. For complementation, a complementation vector containing 1.5 kb promoter region, the *PAL1* gene‐coding region, and the adjacent 0.5 kb downstream region was transformed into the Δ*pal1* mutant. CM plates supplemented with 250 μg/ml hygromycin B (Roche Diagnostics) were used to select the deletion transformants, and CM plates supplemented with 400 μg/ml neomycin (Amresco) were used to select the complementation transformants.

### 4.4 Staining assays

The fungal mycelia incubated in liquid CM were harvested and stained with 10 μg/ml FM4‐64 (Sigma‐Aldrich) at room temperature for 1, 5, 10, or 15 min. For CFW staining, the hyphae and conidia were stained with 10 μg/ml CFW (Sigma‐Aldrich) solution for 10 min. The samples were washed twice with phosphate‐buffered saline (PBS), and observed and photographed under a microscope (Ni90 microscope; Nikon). For glycogen and lipid droplet staining, the spore suspension (10^5^ spores/ml) was inoculated on the hydrophobic plastic cover glass and stained with KI/I_2_ solution (60 mg/ml KI, 10 mg/ml I_2_) for glycogen staining or Nile red solution (50 mM Tris/maleate buffer, 20 mg/ml polyvinylpyrrolidone, 2.5 μg/ml Nile red, pH 7.5) for lipid droplet staining. For vacuole staining, the mycelia were treated with 10 μM prewarmed CMAC (Thermo Fisher Scientific) for 30 min and observed under a microscope.

### 4.5 Infection assay

The rice cultivar *Oryza sativa* ‘CO‐39’ grown for 1 month and the barley cultivar *Hordeum vulgare* ‘E9’ grown for 1 week were used for the virulence test. Conidial suspension (5 × 10^4^ conidia/ml in 0.025% Tween 20) was used to spray plants, which were then incubated under full humidity conditions at 28‐°C. The disease lesions were photographed at 5 dpi.

### 4.6 Subcellular localization

The *PAL1*, *SEP5*, *SEP6*, and *Rab5* genes with native promoters were amplified and ligated into the C‐terminal end of the GFP gene in the vector pKNRG (Tables S2 and S3; Liu et al., 2018). The subsequent vector pKNRG‐PAL1 was transformed into the Δ*pal1* mutant. The constructed vectors pKNRG‐SEP5, pKNRG‐SEP6, and pKNRG‐Rab5 were transformed into wild‐type strain P131 or the Δ*pal1* mutant. Subsequent transformants were used to observe subcellular localization at different developmental stages under a confocal microscope TCS SP8 (Leica Microsystems). To observe the actin ring in appressorium, the pKNRR‐LifeAct vector was constructed and transformed into wild‐type strain P131 and the Δ*pal1* mutant. Transformants with fluorescence were used for observation under the confocal microscope.

### 4.7 Yeast two‐hybrid experiment

The full‐length cDNA of *PAL1* was inserted into vector pGADT7, and the full‐length cDNA of *SLA1* was inserted into vector pGBDKT7. Both vectors were cotransformed into yeast strain AH109. The transformants were selected on plates of SD/−Leu/−Trp and SD/−Leu/−Trp/−His (Clontech), followed by culture at 30°C for 3–4 days to observe the colony growth. The colonies were then transferred to a new SD/−Leu/−Trp/–His plate for staining by 5 μl X‐α‐Gal, followed by incubation at 30°C in darkness for 2 h. The interaction between pGBKT7‐53 and pGADT7‐T was used as the positive control, and the interaction between pGBKT7‐Lam and pGADT7‐T was used as the negative control.

### 4.8 cAMP and IBMX treatment

For determination of cAMP content, the mycelia of different strains were cultured in CM medium for 48 h. The mycelia were collected and treated with liquid nitrogen then freeze‐dried for 16 h. Then cAMP levels were quantified using cAMP Biotrak Enzyme Immunoassay system (Biotrak).

To test the effect of cAMP and IBMX on appressorium formation of the Δ*pal1* deletion mutant, spore suspension (10^5^ spores/ml) was inoculated on a hydrophobic glass slide and then 1 mM cAMP (Macklin) and 2.5 mM IBMX (Solarbio) were added to observe the appressorium formation ratio at different time points.

### 4.9 Pmk1 phosphorylation detection assay by western blotting

Around 200 mg of mycelia was harvested and ground into powder with liquid nitrogen, and resuspended in 1 ml of extraction buffer (10 mM Tris‐HCl pH 7.5, 150 mM NaCl, 0.5 mM EDTA, 0.5% Triton X‐100) with 1 mM phenylmethanesulfonyl fluoride (PMSF) and 10 μl of protease inhibitor cocktail (Sigma). Total proteins were separated by 12% SDS‐PAGE and transferred to nitrocellulose membranes. The Pmk1 protein level was assayed by using anti‐p44/42 MAPK (Erk1/2) antibody (Cell Signaling Technology), and the phosphorylated Pmk1 was assayed by anti‐phospho‐p44/42 MAPK (Erk1/2) (Thr202/Tyr204) antibody (Cell Signaling Technology).

### 4.10 Autophagy assay

The MoAtg8‐GFP construct was transformed into P131 and Δ*pal1*. Subsequent transformants were cultured in liquid minimal medium with nitrogen starvation (MM−N). After 0, 2, or 5 h incubation, the mycelia were harvested for extracting total proteins. A western blot was then performed using total proteins, and the GFP:MoAtg8 fusion protein and free GFP were detected using an anti‐GFP antibody. Protein levels were normalized using an anti‐actin antibody.

### 4.11 Cytorrhysis assay

Appressorium turgor was assessed by dropping conidial suspension (10^5^ conidia/ml) onto a hydrophobic coverslip and incubating it in a moistened chamber at 28°C for 24 h to form mature appressoria. The coverslip was then placed into solution containing different concentrations of PEG 8000 (25%, 30%, 35%, and 40% wt/vol). After 10 min, the percentage of collapsed appressoria was calculated under a microscope. For each experiment, at least 100 conidia were examined and three replicates were performed.

### 4.12 Statistical analysis

Data were subjected to analyses of variance using SPSS v. 19.0 software (IBM). Means were separated using the test of least significant difference (α = 0.05).

## CONFLICT OF INTEREST

The authors declare no conflict of interest exists.

## AUTHOR CONTRIBUTIONS

J.X. and X.L.C. conceived the study. J.X. and X.L.C. designed the experiments. D.C., H.H., W.H., S.Z., M.T., S.X. C.L. X.C., A.H., and M.K. performed the experiments. J.X., X.L.C., D.C., and H.H. analysed data and wrote the manuscript. J.X., X.L.C., J.H., L.Z., and H.L. supervised the project. All authors discussed the results and contributed to the final manuscript.

## Data Availability

The data that support the findings of this study are available from the corresponding author upon reasonable request.
