# Supplementary material for: Endocytic protein Pal1 regulates appressorium formation and is required for full virulence of Magnaporthe oryzae
Source: Mol Plant Pathol. 2021 Oct 12;23(1):133–47. doi: 10.1111/mpp.13149 (PMC8659611; doi:10.1111/mpp.13149)
Supplement: Supplementary file 1 [file MPP-23-133-s008.docx]

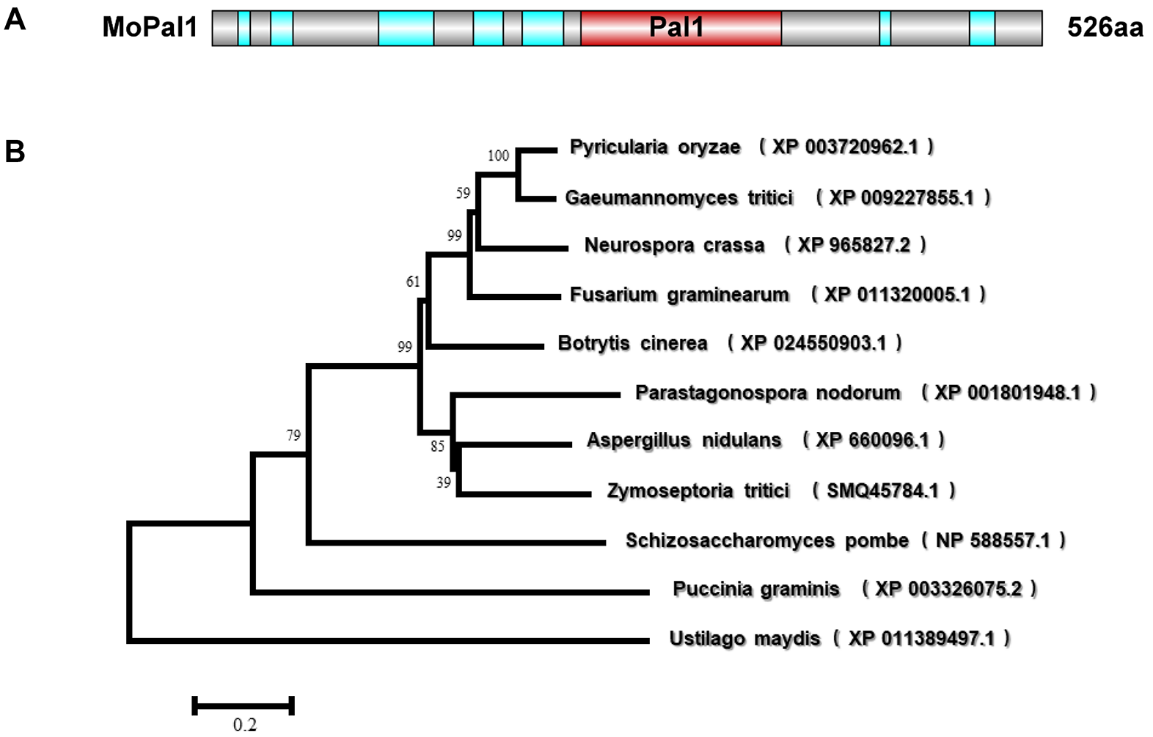


**Fig. S1** **Analyses of Pal1 functional domain and systematic evolution.** (A) Analysis of Pal1 protein structure. Region marked red shows Pal1 domain. Grey fragment means exons and Green part means introns. (B) Systematic evolution analysis of Pal1 among fission yeast and several plant pathogenic pathogens.
