# Supplementary material for: Endocytic protein Pal1 regulates appressorium formation and is required for full virulence of Magnaporthe oryzae
Source: Mol Plant Pathol. 2021 Oct 12;23(1):133–47. doi: 10.1111/mpp.13149 (PMC8659611; doi:10.1111/mpp.13149)
Supplement: Supplementary file 2 [file MPP-23-133-s001.docx]

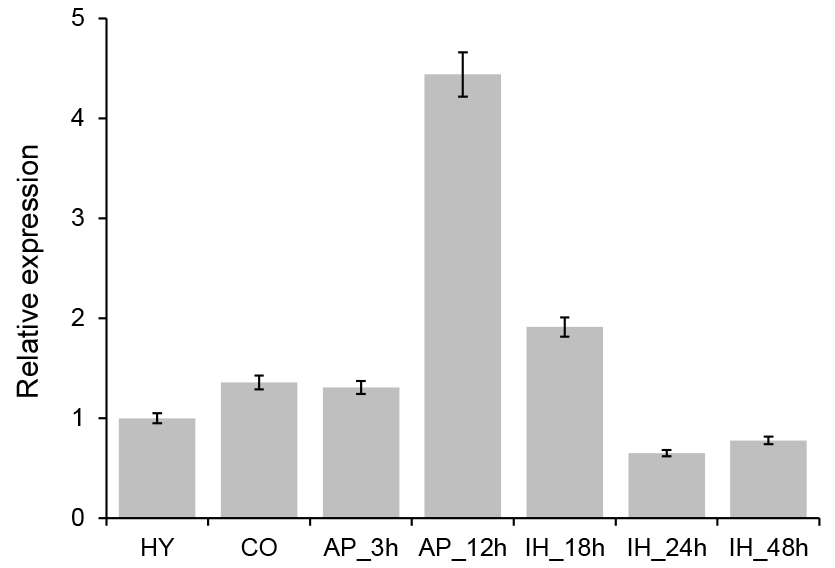


**Fig. S2** **Pal1 expression at stages of hyphae, conidia, appressorium, and infectious hyphae.** HY-hyphae; CO-conidia; AP-3h, appressorium at 3 h; AP-12h, appressorium at 12 h; IH-18h, infectious hyphae at 18 h; IH-24h, infectious hyphae at 24 h; IH-42h, infectious hyphae at 42 h
