# Supplementary material for: Endocytic protein Pal1 regulates appressorium formation and is required for full virulence of Magnaporthe oryzae
Source: Mol Plant Pathol. 2021 Oct 12;23(1):133–47. doi: 10.1111/mpp.13149 (PMC8659611; doi:10.1111/mpp.13149)
Supplement: Supplementary file 3 [file MPP-23-133-s006.docx]

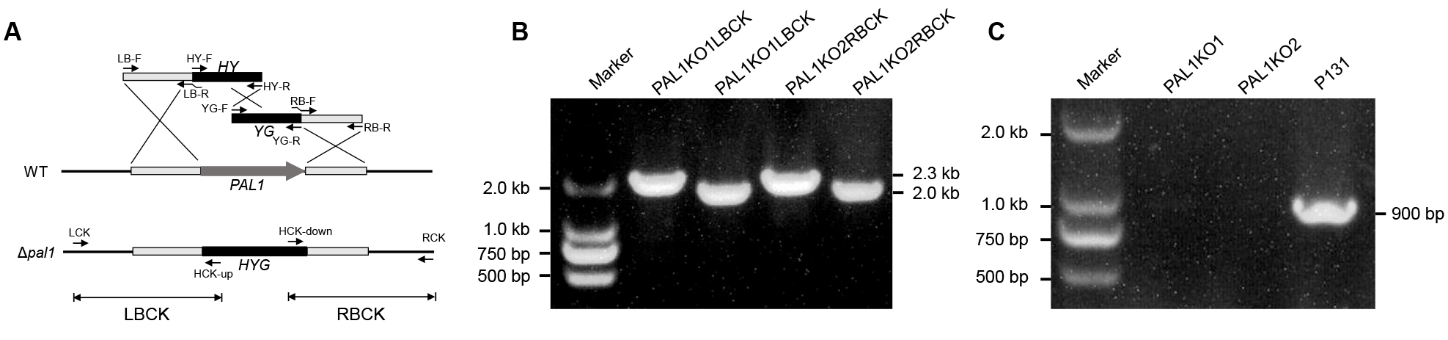


**Fig. S3** **Replacement strategy and confirmation of *PAL1* deletion mutants.** (A) Gene replacement of *PAL1* through a split-marker approach. White bars represent genomic regions upstream and downstream of the *PAL1* coding sequence that was amplified and fused to segments of the hygromycin phosphotransferase (*HYG*) cassette. (B) PCR verification of the flanking sequences besides the replacement fragment by using primer pairs of LCK/HCK-up and RCK/HCK-down. (C) RT-PCR verification by amplifying the *PAL1* fragment in the transformants and the wild-type strain (WT).
