# Supplementary material for: Endocytic protein Pal1 regulates appressorium formation and is required for full virulence of Magnaporthe oryzae
Source: Mol Plant Pathol. 2021 Oct 12;23(1):133–47. doi: 10.1111/mpp.13149 (PMC8659611; doi:10.1111/mpp.13149)
Supplement: Supplementary file 4 [file MPP-23-133-s007.docx]

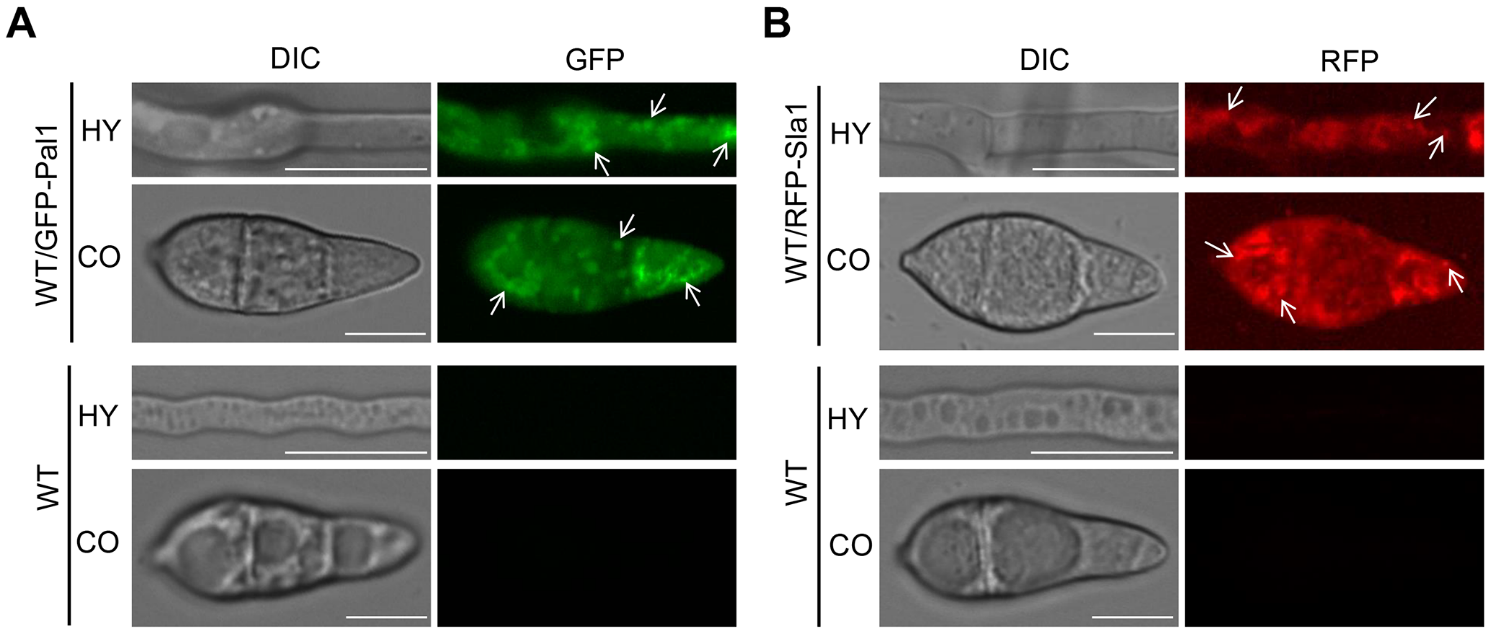


**Fig. S4** Subcellular localization of Pal1 and Sla1 in hyphae, conidia, and appressoria. White arrows indicate the puncta. Bar = 10 μm
