# Supplementary material for: Endocytic protein Pal1 regulates appressorium formation and is required for full virulence of Magnaporthe oryzae
Source: Mol Plant Pathol. 2021 Oct 12;23(1):133–47. doi: 10.1111/mpp.13149 (PMC8659611; doi:10.1111/mpp.13149)
Supplement: Supplementary file 5 [file MPP-23-133-s004.docx]

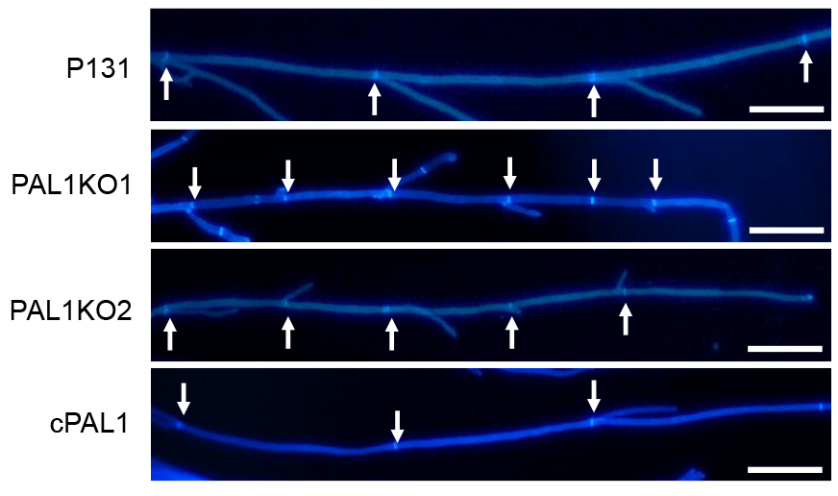


**Fig. S5 Hyphal cells of different strains stained by CFW.** Arrow means cell wall of the mycelium. Bar = 10 μm.
