# Supplementary material for: Endocytic protein Pal1 regulates appressorium formation and is required for full virulence of Magnaporthe oryzae
Source: Mol Plant Pathol. 2021 Oct 12;23(1):133–47. doi: 10.1111/mpp.13149 (PMC8659611; doi:10.1111/mpp.13149)
Supplement: Supplementary file 6 [file MPP-23-133-s009.docx]

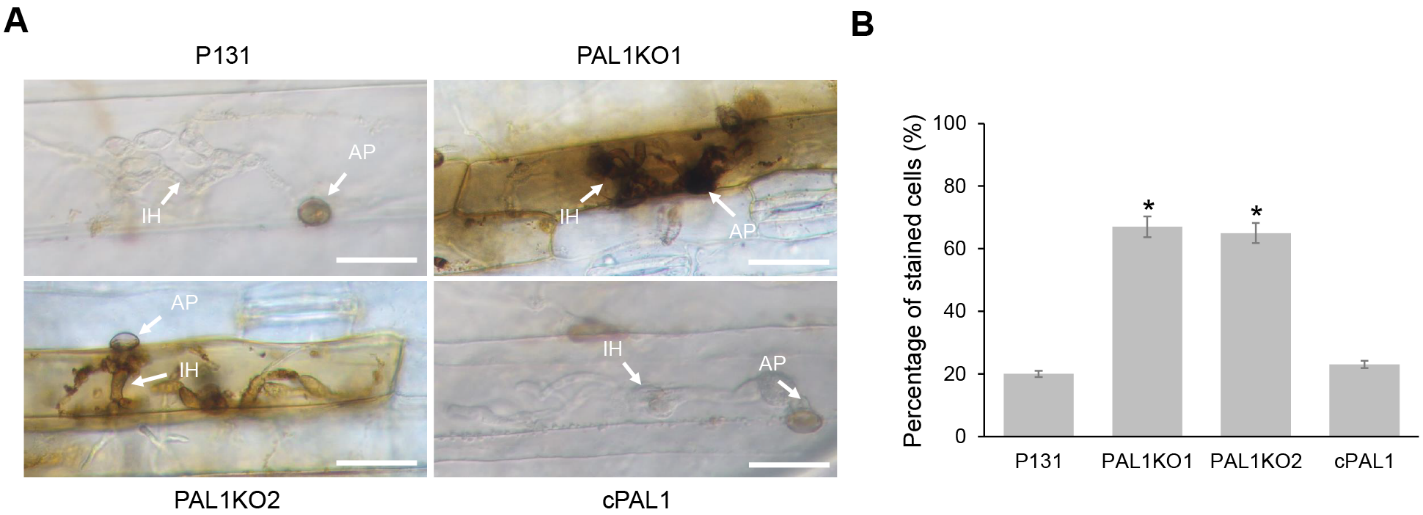


**Fig. S6 Barley cells infected by strains were stained with DAB.** (A) Reactive oxygen species (ROS) produced by barley cells were stained reddish brown by DAB. Bar = 25 μm. (B) Statistical analysis of stained barley cells infected by P131, PAL1KO1, PAL1KO2, and cPAL1. Significant difference was marked with single asterisk (*P* < 0.05).
