# Supplementary material for: Endocytic protein Pal1 regulates appressorium formation and is required for full virulence of Magnaporthe oryzae
Source: Mol Plant Pathol. 2021 Oct 12;23(1):133–47. doi: 10.1111/mpp.13149 (PMC8659611; doi:10.1111/mpp.13149)
Supplement: Supplementary file 7 [file MPP-23-133-s002.docx]

**Table S1 Fungal strains used in this study.**

| **Strains** | **Genotypes** | **References** |
| --- | --- | --- |
| P131 | A wild-type isolate of *M. oryzae* | Peng and Shishiyama (1988) |
| PAL1KO1, PAL1KO2 | *PAL1*deletion mutants of P131, Δ*pal1*. | This study |
| cPAL1 | Complement strain of Δ*pal1*, Δ*pal1/PAL1* | This study |
| PAL1G | P131 transformed by *eGFP-PAL1* fusion construct, WT/GFP:PAL1. | This study |
| SLA1R | P131 transformed by *eRFP-SLA1* fusion construct, WT/RFP:SLA1. | This study |
| LifeActR | P131 transformed by *eRFP-LifeAct* fusion construct, WT/RFP: LifeAct. | This study |
| pal1/LifeAct | Δ*pal1* transformed by *eRFP-LifeAct* fusion construct, Δ*pal1*/RFP: LifeAct. | This study |
| pal1/*GFP-PAL1­-*LifeAct | Δ*pal1* co-transformed by *eGFP-PAL1* and *eRFP-LifeAct*, Δ*pal1*/ *eGFP-PAL1-*RFP: LifeAct. | This study |
| Sep5G | P131 transformed by *eGFP-SEP5* fusion construct, WT/GFP:SEP5. | This study |
| Sep6G | P131 transformed by *eGFP-SEP6* fusion construct, WT/GFP:SEP6. | This study |
| Rab5G | Δ*pal1* transformed by *eGFP-Rab5* fusion construct, WT/GFP:SEP6. | This study |
| pal1/Sep5G | Δ*pal1* transformed by *eGFP-SEP5* fusion construct, Δ*pal1*/GFP: SEP5. | This study |
| pal1/Sep6G | Δ*pal1* transformed by *eGFP-SEP6* fusion construct, Δ*pal1*/GFP: SEP6. | This study |
| ATG8G | P131 transformed by *eGFP-ATG8* fusion construct, WT/GFP:ATG8. | This study |
| pal1/ATG8G | Δ*pal1* transformed by *eGFP-ATG8* fusion construct, Δ*pal1*/GFP: ATG8. | This study |

Peng, Y.L. & Shishiyama, J. (1988) Temporal sequence of cytological events in rice leaves infected with *Pyricularia oryzae*. *Canadian Journal of Botany*, 66, 730–735.
