# Supplementary material for: Endocytic protein Pal1 regulates appressorium formation and is required for full virulence of Magnaporthe oryzae
Source: Mol Plant Pathol. 2021 Oct 12;23(1):133–47. doi: 10.1111/mpp.13149 (PMC8659611; doi:10.1111/mpp.13149)
Supplement: Supplementary file 8 [file MPP-23-133-s003.docx]

**Table S2 Plasmids used in this study.**

| **Names** | **Descriptions** |
| --- | --- |
| pKN | Vector used to construct complementation vectors and other vectors; with the *NPTII* gene as a selective marker inserted into pKS^+^ (Yang et al., 2010). |
| pKN-*PAL1* | *PAL1* complementation vector; *PAL1* gene containing 1.5 kb promoter and 0.5 kb terminator regions were amplified and inserted into pKN. |
| pKNRG | Vector used to construct vectors to constitutively express selected genes; with the fungal constitutive promoter RP27 (Yang et al., 2010). |
| pKNRG-*PAL1* | Vector for sub-cellular localization of PAL1 protein; coding region of *PAL1* was cloned into vector pKNRG. |
| pKNRG-*Rab5* | Vector for sub-cellular localization of Rab5 protein; coding region of *Rab5* was cloned into vector pKNRG. |
| pKNRG-*SEP5* | Vector for sub-cellular localization of SEP5 protein; coding region of *SEP5* was cloned into vector pKNRG. |
| pKNRG-*SEP6* | Vector for sub-cellular localization of SEP6 protein; coding region of *SEP6* was cloned into vector pKNRG. |
| pKNRR-*SLA1* | Vector for sub-cellular localization of SLA1 protein; coding region of *SLA1* was cloned into vector pKNRR. |
| pKNRR-*LifeAct* | Vector for sub-cellular localization of LifeAct protein; coding region of *LifeAct* was cloned into vector pKNRR. |
| pKNRG-*ATG8* | Vector for sub-cellular localization of ATG8 protein; coding region of *ATG8* was cloned into vector pKNRG. |

Yang, J., Zhao, X., Sun, J., Kang, Z., Ding, S., Xu, J.R and Peng, Y.L. (2010) A novel protein Com1 is required for normal conidium morphology and full virulence in *Magnaporthe oryzae*. *Molecular* *Plant-Microbe* *Interactions* 23, 112-123.
