# Supplementary material for: Endocytic protein Pal1 regulates appressorium formation and is required for full virulence of Magnaporthe oryzae
Source: Mol Plant Pathol. 2021 Oct 12;23(1):133–47. doi: 10.1111/mpp.13149 (PMC8659611; doi:10.1111/mpp.13149)
Supplement: Supplementary file 9 [file MPP-23-133-s005.docx]

**Table S3 Primers that used in this study.**

| **Primers** | **Sequences (5'–3')** |
| --- | --- |
| HPT-LCK | GACAGACGTCGCGGTGAGTT |
| HPT-RCK | TCTGGACCGATGGCTGTGTAG |
| HPT-F1 | CTCCGACCTGATGCAGCTCT |
| HPT-R1 | CTCGCTCCAGTCAATGACC |
| HYG-LB | ACCTCCACTAGCTCCAGCCAAG |
| HYG-RB | GAATAGAGTAGATGCCGACCGGG |
| PAL1LBCK | GATGGCCTCCTGTCTACTACGT |
| PAL1LB-F | TGATGGCCTCCTGTCTACTA |
| PAL1LB-R | ACCTCCACTAGCTCCAGCCAAGAGATCTTGCT CCGCAAAA |
| PAL1RB-F | GAATAGAGTAGATGCCGACCGGGACTGACAAAGCATTC |
| PAL1RB-R | GCTGCGAGATCTTGCCAC |
| PAL1RBCK | TTGAAGAGTCCCACAGTCCA |
| PAL1-F  PAL1-R | ATGTCATCGG GAATGCATCC  GGATGCCTCCGCTCGAAGTA |
| PAL1GFP-F | GAGGTCGACGGTATCGATAAGCTTGTATTGACACGCATTGAGAC |
| PAL1GFP-R | AGCACCTCTAGAACTAGTGGATCCGGCTGCGGTACCCGGGGCCG |
| SLA1RFP-F | GGTATCGATAAGCTTATGGGCTTCATCGGCGTCTA |
| SLA1RFP-R | AATCCTTCTTGGATCCTTAGAAGCCAAATGGATTCT |
| ATG8-GFP-F | ATGGACGAGCTGTACAAGGGATCCATGCGCTCCAAGTTCAAGGA |
| ATG8-GFP-R | ATGGACGAGCTGTACAAGGGATCCATGCGCTCCAAGTTCAAGGA |
| Lifeact-RFP-F | TTTCTCGAGATGGCATCCACGGGCGTC |
| Lifeact-RFP-R | TTTCTCGAGATGGCATCCACGGGCGTC |
| SEP5GFP-F | TTCGAATTCCAAATTGTGAAATTGCAT |
| SEP5GFP-R | TTTGGATCCCTCGGTAGTAGTGGTTTC |
| SEP6GFP-F | TTCAAGCTTGGGTTGGTGGGTGGGTGC |
| SEP6GFP-R | TTAGGATCCACCCGAAACTACCATTAAAC |
| PAL1PKN-F | GTCGACGGTATCGATAAGCT TGTATTGACACGCATTGAGAC |
| PAL1PKN-R | TAATCTAGAACTAGTGGATCCATGACGAATGTAGCGCATTA |
| SLA1PGPBKT7-F | ATGGCCATGGAGGCCGAATTCATGGGCTTCATCGGCGTCTA |
| SLA1PGPBKT7-R | GGCCGCTGCAGGTCGACGGATCCTTAGAAGCCAAATGGATTCT |
| PAL1PGADT7-F | GGTGGGCATCGATACGGGATCCATGTCATCGGGAATGCATCC |
| PAL1PGADT7-R | TGCAGCTCGAGCTCGATGGATCCCTAGGCTGCGGTACCCGGGG |
